# Comprehensive clinical evaluation of TomoEQA for patient-specific pre-treatment quality assurance in helical tomotherapy

**DOI:** 10.1186/s13014-022-02151-x

**Published:** 2022-11-07

**Authors:** Changhwan Kim, Min Cheol Han, Young Kyu Lee, Han-Back Shin, Hojin Kim, Jin Sung Kim

**Affiliations:** 1 Department of Radiation Oncology, Yonsei Cancer Center, Yonsei University College of Medicine, 50-1 Yonsei-ro, Seodaemun-gu, Seoul, 03722 Republic of Korea; 2 Department of Radiation Oncology, Seoul St. Mary’s Hospital, College of Medicine, The Catholic University of Korea, 222 Banpo-daero, Seocho-gu, Seoul, 06591 Republic of Korea

**Keywords:** Helical tomotherapy, Patient-specific pre-treatment quality assurance, Exit detector

## Abstract

**Background:**

Based on a previous study on the feasibility of TomoEQA, an exit detector-based patient-specific pre-treatment quality assurance (QA) method for helical tomotherapy, an in-depth clinical evaluation was conducted.

**Methods:**

Data of one hundred patients were analyzed to evaluate the clinical usefulness of TomoEQA for patient-specific pre-treatment QA in comparison with the conventional phantom-based method. Additional investigations were also performed under unusual measurement conditions to validate the off-axis region. In addition to the clinical evaluation of TomoEQA, a statistical analysis was conducted to determine the plan parameters that affect the pass/failure results of pre-treatment QA.

**Results:**

The average and standard deviations of the gamma passing rate and point dose error for TomoEQA were comparable to those of the conventional QA method. For TomoEQA, the average values of the gamma passing rate and point dose error were 96.32% (standard deviation (1 sigma) = 3.94; 95% confidence interval (CI), 95.55 to 97.09) and − 1.12% (standard deviation (1 sigma) = 1.04; CI, − 1.32 to − 0.92), respectively. For the conventional QA method, the average values of the gamma passing rate and point dose error were 95.95% (standard deviation (1 sigma) = 4.35; 95% confidence interval (CI), 95.10 to 96.80) and − 1.20% (standard deviation (1 sigma) = 1.61; CI, − 1.52 to − 0.88), respectively. Further experiments on the off-axis region demonstrated that TomoEQA can provide accurate results for 3D dose analysis, which is inherently difficult in the conventional QA method. Through a statistical analysis based on the results of TomoEQA, it was validated that the total fraction (Total Fx), monitor units, beam-on-time, leaf-of-time below 100 ms, and planning target volume diameter were statistically significant for the pass/failure of the pre-treatment QA results.

**Conclusions:**

TomoEQA is a clinically beneficial alternative to the conventional phantom-based QA method.

**Supplementary Information:**

The online version contains supplementary material available at 10.1186/s13014-022-02151-x.

## Introduction

Patient-specific pre-treatment quality assurance (QA) is a crucial part of radiotherapy. It ensures accurate delivery of the planned dose under actual treatment conditions of a linear accelerator (LINAC). Because radiation beams are irradiated through complex combinations of each part of the treatment system, such as the multi-leaf collimator (MLC) positions and gantry rotation, even a small amount of uncertainty in these variables can have a significant impact on treatment [1–3]. Therefore, patient-specific pretreatment QA has gained considerable attention. A typical QA method measures 2D dose distribution and the point dose in a water-equivalent phantom using a radiochromic film and an ionization chamber. In the case of helical tomotherapy (HT), where a ring gantry-type LINAC delivers radiation in the helical beam trajectory, a cylindrical Virtual Water™ phantom provided by Accuray Inc. (Sunnyvale, CA, USA) is mainly employed for patient-specific pre-treatment QA. This water-equivalent phantom dedicated to HT is also known as a cheese phantom and is designed to accommodate film insertion in the coronal plane for 2D dose distribution. Furthermore, an Exradin A1SL ionization chamber (Standard Imaging Inc., Middleton, WI, USA) is placed above the film plane for point-dose measurement.

Phantom-based QA is simple to apply in a clinical setting and provides the benefits of measuring the actual dose delivered to the phantom. However, it has several inherent limitations. One of the significant drawbacks is that it may not accurately reflect the tissue heterogeneity of the patient because the treatment plan is delivered to a homogeneous phantom. In addition, careful management of many physical factors that adversely affect the measurement accuracy, including the setup position of the phantom, chamber, and film, is required. Furthermore, simultaneously measuring the point dose at several spots is practically difficult. Although cheese phantoms offer multiple measurement locations, they are not easy to apply in clinical practice owing to the requirement of several chambers or the need to perform multiple measurements with one chamber while changing the position. It is difficult to verify the overall 3D dose distribution because only the 2D planar dose distribution measurement is inherently available.

To overcome these limitations, various measurement devices and appropriate patient-specific QA methods have been proposed. Two-dimensional array QA devices, such as MatriXX (IBA Dosimetry, Schwarzenbruck, Germany) and MapCHECK (SNC, Melbourne, FL, USA), are appropriate for clinical use as only the device must be installed. However, only 2D planar dose distribution can be measured using such devices. Moreover, another inherent problem is the verification of composite rotational dose distributions using a single-plane array owing to the angular dependency of the system. Delta4 (ScandiDos, Madison, WI, USA; biplanar diode array) and ArcCHECK (SNC, Melbourne, FL, USA; cylindrical diode array) are more innovative devices that are capable of semi-3D dosimetry, and the dose distribution information can be preserved regardless of the beam incidence angle. However, the measurement errors of such devices tend to be underestimated because data post-processing procedures, including interpolation and setup error correction, are required [4–8]. To compensate for the limitations of these existing QA methods, TomoEQA, a log-based patient-specific pre-treatment QA method dedicated to HT, was proposed in our previous work [9]. It employs actual leaf-open-time (LOT) data acquired from the exit detector of HT as a type of machine log data to calculate the 3D dose distribution. The workflow of this method is similar to that of existing log-based QA methods, which are widely used in general LINAC systems [10–17]. However, unlike a general LINAC, the HT system does not provide sufficient machine log data; therefore, the novelty of TomoEQA is that the actual movement of the MLC is obtained from the exit detector. The actual LOT is indirectly derived from the sinogram data obtained from the exit detector while delivering the treatment plan in air without a phantom. Subsequently, the actual LOT is updated in the RT-plan file, and the modified file is utilized to calculate the 3D dose in the secondary treatment planning system (TPS) (Mobius3D in this study).

The feasibility of TomoEQA has been validated in a previous study. In this study, an in-depth clinical evaluation of TomoEQA was performed using the data of one hundred patients. The results of TomoEQA were compared with those of phantom-based QA with a cheese phantom, a current clinical routine in our institute. The existing method can only check the 2D planar dose distribution, whereas TomoEQA can be extended to 3D dose distribution, thus enabling overall dose comparison and analysis. Therefore, the critical performance of QA and the results of TomoEQA were compared with those of the existing method to determine whether the differences affect the detection of clinically unacceptable plans. Additional measurements of phantom-based QA were also conducted, including film measurements on different 2D planes (i.e., sagittal plane) or off-axis point measurements, to evaluate other cases where failures are detected only when TomoEQA is used. We also performed statistical analyses to determine the relationships between plan parameters and the pass/failure trends of patient-specific pre-treatment QA using TomoEQA.

## Materials and methods

### TomoEQA

**Fig. 1:**
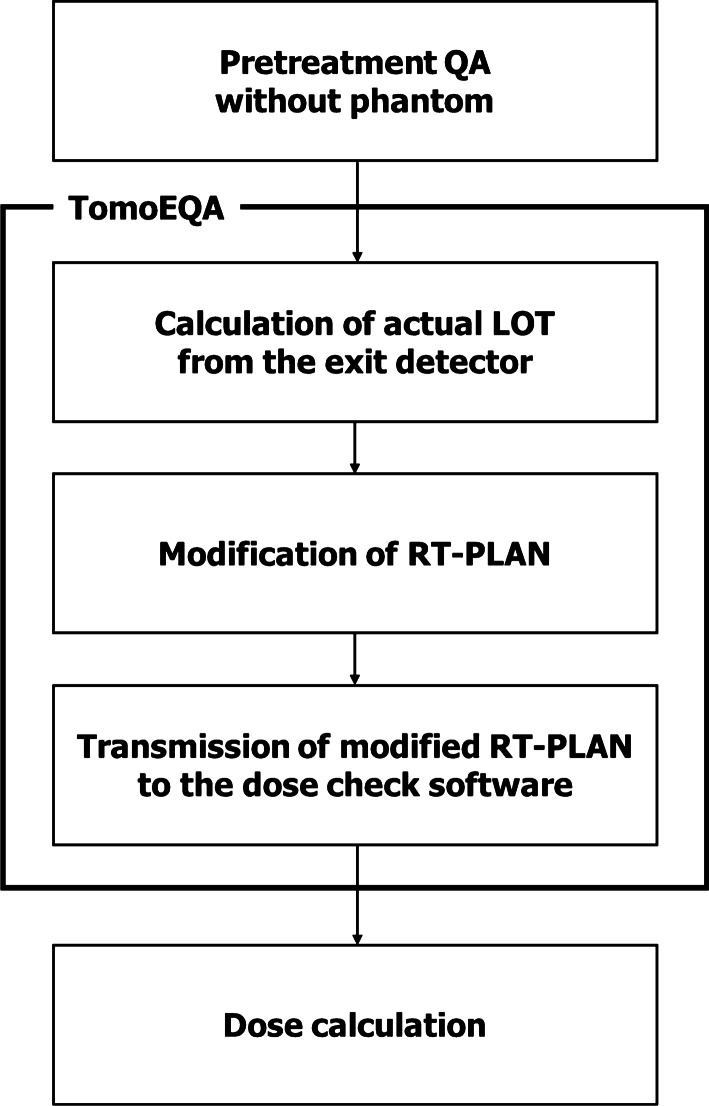
Overall workflow of TomoEQA

The workflow of TomoEQA consists of three main steps: calculation of the actual LOT from exit detector data, modification of the RT-plan file, and transmission of the modified RT-plan to the secondary dose check software. The QA plan should be delivered in advance without any object, and the relevant collected detector data should be utilized for TomoEQA. The first step involves conversion of the data domain from the exit detector to the MLC using leaf-to-channel mapping, as described in previous studies [18, 19]. A correction process is also applied through iterative deconvolution to consider the physical characteristics of the peripheral leaves, which are the primary origin of beam scattering and penumbra. A subsequent normalization process is applied to match the amplitude of the signal affected by detector sensitivity and jaw size. The detector data obtained under half of the planned jaw size are superseded by planned data to compensate for insufficient detector data in dynamic jaw delivery in HT [18]. The second step involves implementing the processed data in a DICOM RT-plan file. After conversion to the ratio of the LOT to the rotation period, the original information embedded in the “Tomo Projection Sinogram Data” attribute is replaced with the converted data. The last step exports a revised DICOM RT-plan to the secondary dose check software and recalculates the dose accordingly. In this study, Mobius3D was used as the secondary dose check software. The dose calculated through these procedures indicates the actual delivery conditions of the treatment system, thereby enabling patient-specific pre-treatment QA. Because TomoEQA is based on the secondary TPS, which is independent of the primary TPS, cross-validation for dose calculation is also feasible. Three-dimensional dose evaluation based on heterogeneous patient geometry is an additional advantage over existing phantom-based methods. The overall workflow of TomoEQA is shown in Fig. 1. Details of this method are available in our previous work [9].

### Comparative study

The results of TomoEQA were compared with those of the conventional method to perform an in-depth clinical evaluation of TomoEQA. In the case of TomoEQA, 3%/3 mm criteria and a 10% dose threshold were employed for an absolute global 3D gamma evaluation based on the default setting provided by Mobius3D. For the point dose comparison, a spherical structure with a radius of 0.25 cm was generated in the high-dose regions of the planning target volume (PTV) with low-dose gradients, similar to that in the conventional phantom-based method. The mean dose over this structure was calculated and compared with the reference dose of the TPS. In the conventional phantom-based approach, the 2D dose distribution and point dose were measured using an EBT3 film and ionization chamber in the cheese phantom, respectively. The films were scanned using a VIDAR Dosimetry PRO™ digitizer (Vidar Systems Corporation, Hendon, Virginia) and analyzed using RIT film dosimetry software (Radiological Imaging Technology, Colorado Springs, CO).

For both methods, the same pass/failure criteria were applied such that the percentage of passing gamma (gamma passing rate, GPR) with 3%/3 mm tolerances would be higher than 90%, and the absolute point dose error would be less than 5%. For a more detailed analysis, the gamma passing rate and point dose error were subdivided into four levels. Further classification was performed by defining the higher value of the two scores as the total QA score, as presented in Table 1.

**Table 1:** Scoring total QA, gamma passing rate, and point dose error of pretreatment QA

Total QA score		
Max (Gamma passing rate score, Point dose error score)		
	Gamma passing rate score	Point dose error score
Pass		
Grade 1	98% ≤ PR	AD ≤ 1%
Grade 2	95% ≤ PR < 98%	1% < AD ≤ 2.5%
Grade 3	90% ≤ PR < 95%	2.5% < AD ≤ 5%
Failure		
Grade 4	PR < 90%	5% < AD

*PR* passing rate, *AD* absolute dose error

In cases where the QA result of the existing method was satisfied but TomoEQA did not meet the criteria, additional measurements of the conventional method were performed, including film measurements on different 2D planes and off-axis point measurements for further verification.

### Experimental conditions

One hundred treatment plans of patients who underwent radiotherapy with HT at our institute between May 2021 and June 2021 were included in this study to evaluate the clinical usefulness of TomoEQA as a patient-specific pre-treatment QA in comparison with the conventional phantom-based method. All treatment plans were generated using Accuray Precision TPS (version 6.1.0.3.11) of Radixact (Accuray Inc., Sunnyvale, CA, USA). The calculation grid was set to “fine,’ translating into a voxel size of approximately 1.0 mm. All the cases, except the one with a jaw size of 1.05 cm, were generated and delivered using the dynamic jaw technique. This study was approved by the Institutional Review Board (IRB) of Yonsei University Hospital (approval number: 4-2022-0392). All data were fully anonymized before the investigators accessed them. The requirement for written informed consent was waived by the IRB because of the retrospective nature of the study. The characteristics of the patients, including the mean and standard deviation of the individual plan parameters, are summarized in Table 2. Patient data were classified into nine clinical categories according to the treatment site.

**Table 2:** Summary of patient characteristics, including the plan parameters used in this study

Treatment Sites		Total Fx	Fx Dose [Gy]	MU	Pitch	Jaw Size [mm]	BOT [s]	MF	Mean LOT [ms]	LOT below 100 ms [%]	Offset [mm]	PTV diameter [mm]	PTV length [mm]	Dynamic fraction* [%]
Brain (17)	Average	17.35	2.35	5278.26	0.33	25.08	319.87	2.06	133.47	28.35	29.05	125.14	122.24	9.37
	SD	9.70	0.44	2314.38	0.10	0.00	118.89	0.30	37.58	12.00	18.78	61.11	153.76	3.42
Head and Neck (26)	Average	24.92	2.16	4012.87	0.39	24.51	252.80	2.13	144.12	31.99	40.71	71.33	60.58	10.44
	SD	8.46	0.72	1295.31	0.07	2.88	108.36	0.21	20.49	9.60	16.44	31.10	33.71	6.19
Pelvis (8)	Average	10.13	4.65	6111.03	0.21	34.59	329.71	1.87	194.55	37.56	65.74	106.38	104.13	18.67
	SD	8.64	1.73	2946.70	0.06	13.13	164.86	0.35	38.09	10.18	35.32	64.08	89.28	4.37
Prostate (21)	Average	22.67	2.17	5325.15	0.37	26.29	296.47	2.16	171.65	33.92	14.23	76.66	55.00	10.35
	SD	5.00	0.90	1504.89	0.07	5.53	90.95	0.32	45.86	9.75	14.27	34.19	57.63	2.76
Rectum (6)	Average	19.17	2.53	6647.67	0.26	25.08	431.68	2.06	172.18	24.86	79.08	106.86	102.17	10.49
	SD	9.17	1.30	2689.07	0.04	0.00	158.80	0.21	74.05	7.50	91.33	33.63	42.43	5.57
Spine (4)	Average	8.75	3.88	5902.75	0.24	37.76	308.55	1.94	185.67	35.15	27.94	106.71	191.75	20.92
	SD	7.50	1.65	2091.82	0.13	14.64	135.00	0.21	43.55	8.17	14.10	31.53	294.49	16.84
Cervix (9)	Average	22.56	2.26	4972.82	0.38	44.80	274.15	2.05	170.61	30.54	10.43	173.73	196.11	14.59
	SD	7.33	1.40	1847.18	0.11	11.18	80.76	0.19	25.88	11.56	5.42	45.54	97.93	5.08
Large (7)	Average	15.00	2.34	8232.45	0.33	46.82	318.47	2.27	152.00	33.79	83.69	166.93	407.43	9.62
	SD	5.77	1.25	2360.62	0.12	9.59	112.00	0.55	42.23	17.61	102.91	64.64	299.69	7.95
Others (2)	Average	15.00	3.50	3927.33	0.32	37.76	298.30	1.90	210.56	32.30	55.23	104.80	93.00	18.89
	SD	14.14	2.12	1223.38	0.15	17.93	22.83	0.33	39.34	1.63	10.95	7.14	63.64	2.50
Total (100)	Average	19.88	2.53	5285.16	0.34	30.01	299.92	2.09	159.73	31.92	37.54	104.53	118.24	11.81
	SD	9.11	1.24	2180.83	0.10	10.37	117.59	0.30	42.29	10.82	43.03	55.87	150.90	6.47

*SD* standard deviations, *Fx* fraction, *BOT* beam on time, *LOT* leaf open time, *Large* large fields including cranial spinal irradiation or treatment fields with lymph nodes

^*^Dynamic fraction is the ratio of the incomplete projections that were replaced with planned data to compensate for insufficient detector data from the dynamic jaw technique

### Statistical analysis

IN addition to the clinical evaluation of TomoEQA, statistical analysis was conducted to investigate the plan parameters that affect the pass/failure results of pre-treatment QA for HT. This statistical analysis is based on the underlying expectation that the results of patient-specific pre-treatment QA using TomoEQA are significantly reliable as they provide 3D dose evaluation based on heterogeneous patient geometry. Therefore, meaningful outcomes, the correlation between the plan parameters, and the pass/failure results can be derived from them. We compared the *p* value using Welch’s t-test and evaluated whether there was a significant difference between the pass and failure groups according to the individual plan parameters. The sample sizes of the two groups were not comparable; hence, it was determined that a precise statistical analysis may not be possible. Therefore, the analysis in this study was performed by dividing the significance level into three steps in terms of the *p *value: a *p *value of < 0.01 is considered as “highly significant,” a *p *value of < 0.05 is considered as “statistically significant,” and a *p *value of < 0.1 is considered as “can be significant,” as summarized in Table 3 [20]. The statistical analyses were performed using MATLAB R2021a (MathWorks, Natick, MA, USA).

**Table 3:** Level of significance for a range of *p *values

*p *value range	Description on significance level
[0,0.01]	Highly significant
(0.01,0.05]	Statistically significant
(0.05,0.1]	Can be significant

## Results

### Comparison of TomoEQA and conventional QA

Pre-treatment QA was performed on one hundred patients using the conventional phantom-based and TomoEQA methods. Ninety-nine patients satisfied the pass/failure criteria for the conventional QA method, and only one case with a large field failed to meet the criteria. The average values of the gamma passing rate and point dose error were 95.95% (standard deviation (1 sigma) = 4.35; 95% confidence interval (CI), 95.10 to 96.80) and − 1.20% (standard deviation (1 sigma) = 1.61; CI, − 1.52 to − 0.88), respectively. In contrast, ninety-one patients satisfied the pass/failure criteria for TomoEQA, and nine cases failed to meet the criteria. The failed cases include three involving the brain, two involving the head and neck, two involving the pelvis, and two involving a large field. The average values of the gamma passing rate and point dose error were 96.32% (standard deviation (1 sigma) = 3.94; 95% confidence interval (CI), 95.55 to 97.09) and − 1.12% (standard deviation (1 sigma) = 1.04; CI, − 1.32 to − 0.92), respectively. The failure rate increased from 1 to 9%; however, the average and standard deviation of the gamma passing rate and point dose error were comparable in both pre-treatment QA methods. Table 4 summarizes the pre-treatment QA results for each treatment site performed using the conventional QA and TomoEQA methods.

**Table 4:** Pre-treatment QA results for each treatment site performed using the conventional and TomoEQA methods

Treatment sites											
		Brain (17)	Head and Neck (26)	Pelvis (8)	Prostate (21)	Rectum (6)	Spine (4)	Cervix (9)	Large (7)	Others (2)	Total (100)
Conventional QA											
Pass		17	26	8	21	6	4	9	6	2	99
Failure		0	0	0	0	0	0	0	1	0	1
Gamma (%)	Average	96.61	96.31	95.59	96.53	98.15	97.75	94.24	90.63	97.20	95.95
	SD	2.95	2.85	3.68	2.48	1.02	2.32	3.05	12.44	0.85	4.35
PD (%)	Average	− 1.73	− 1.65	− 1.38	− 0.32	− 1.30	− 0.48	− 0.70	− 1.61	− 1.12	− 1.20
	SD	1.26	1.86	2.17	0.87	1.54	1.66	1.10	2.37	0.28	1.61
TomoEQA											
Pass		14	24	6	21	6	4	9	5	2	91
Failure		3	2	2	0	0	0	0	2	0	9
Gamma (%)	Average	94.38	97.22	94.81	98.40	95.28	98.53	97.18	92.31	94.15	96.32
	SD	3.69	2.12	7.76	1.10	3.52	1.31	1.61	7.23	3.75	3.94
PD (%)	Average	− 0.81	− 0.70	− 0.90	− 2.68	− 2.53	0.81	− 1.08	1.24	− 1.87	− 1.12
	SD	1.47	2.14	2.93	0.96	1.18	1.03	0.66	2.29	4.00	2.04

*SD* standard deviations, *PD* point dose error

**Fig. 2:**
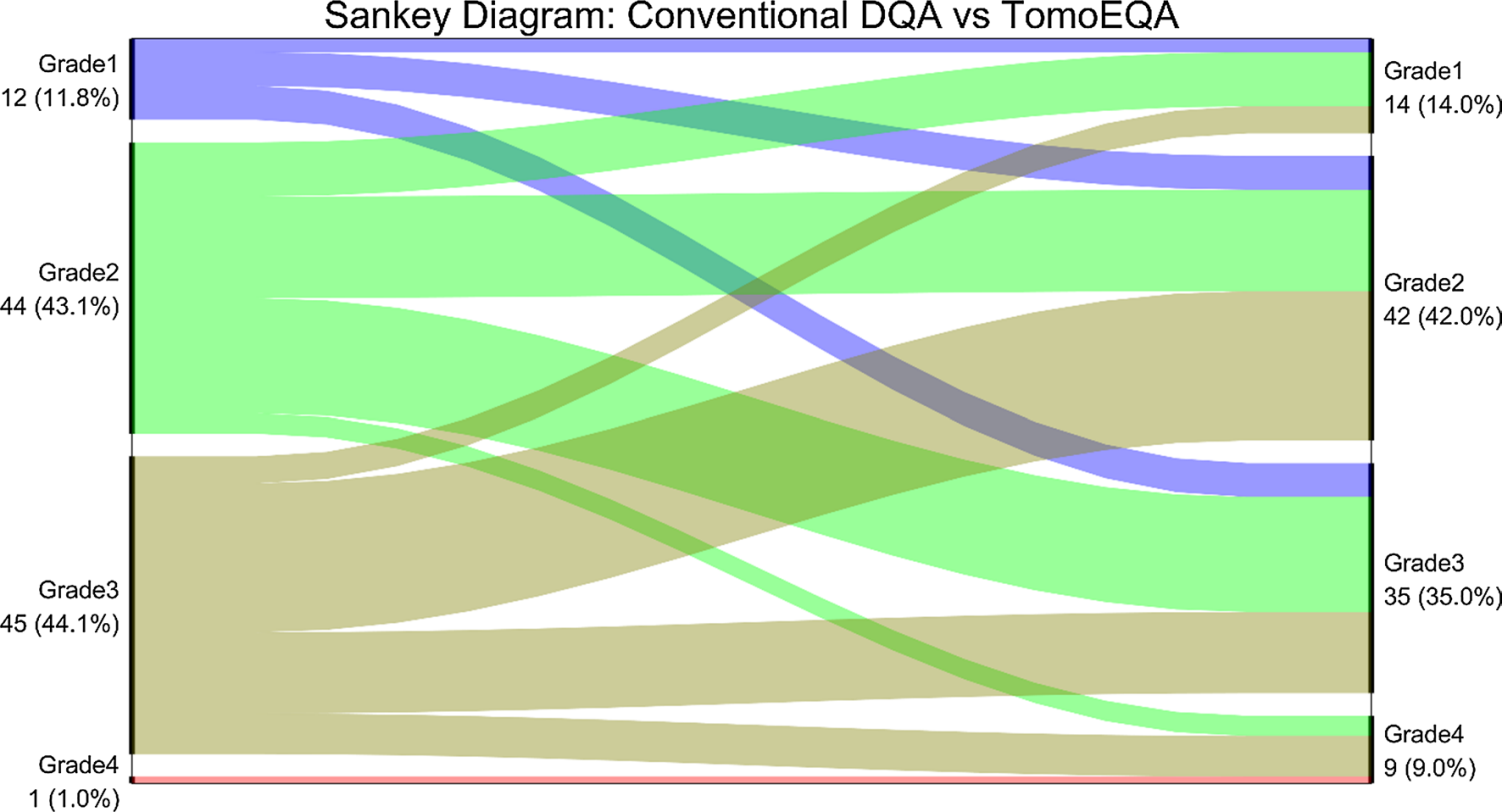
Sankey diagram of the pre-treatment QA results using the conventional QA and TomoEQA methods

A Sankey diagram analysis was used (Fig. 2) based on the total QA score to visualize the relationship between the results of the conventional QA and TomoEQA methods. Although the detailed grade scores were slightly different, most of the cases that satisfied the pass/failure criteria in the conventional QA method also met the criteria in TomoEQA. One case with grade 4, which failed in the conventional QA method, also failed in TomoEQA. In contrast, eight cases satisfied the criteria in the conventional QA method but failed in TomoEQA. Three cases with grade 2 and five cases with grade 3 in the conventional QA method did not meet the criteria in TomoEQA; these require further investigation. Most of the failures originated from hot or cold spots near the off-axis, and additional measurements were conducted to validate the TomoEQA results. Based on the location of the hot and cold spots confirmed by TomoEQA, we conducted conventional phantom-based measurements by changing the position of the chamber or the plane on which the film was placed. Details of the experiments and relevant results are described in the next section.

### Further investigation of failed plans on TomoEQA

**Fig. 3:**
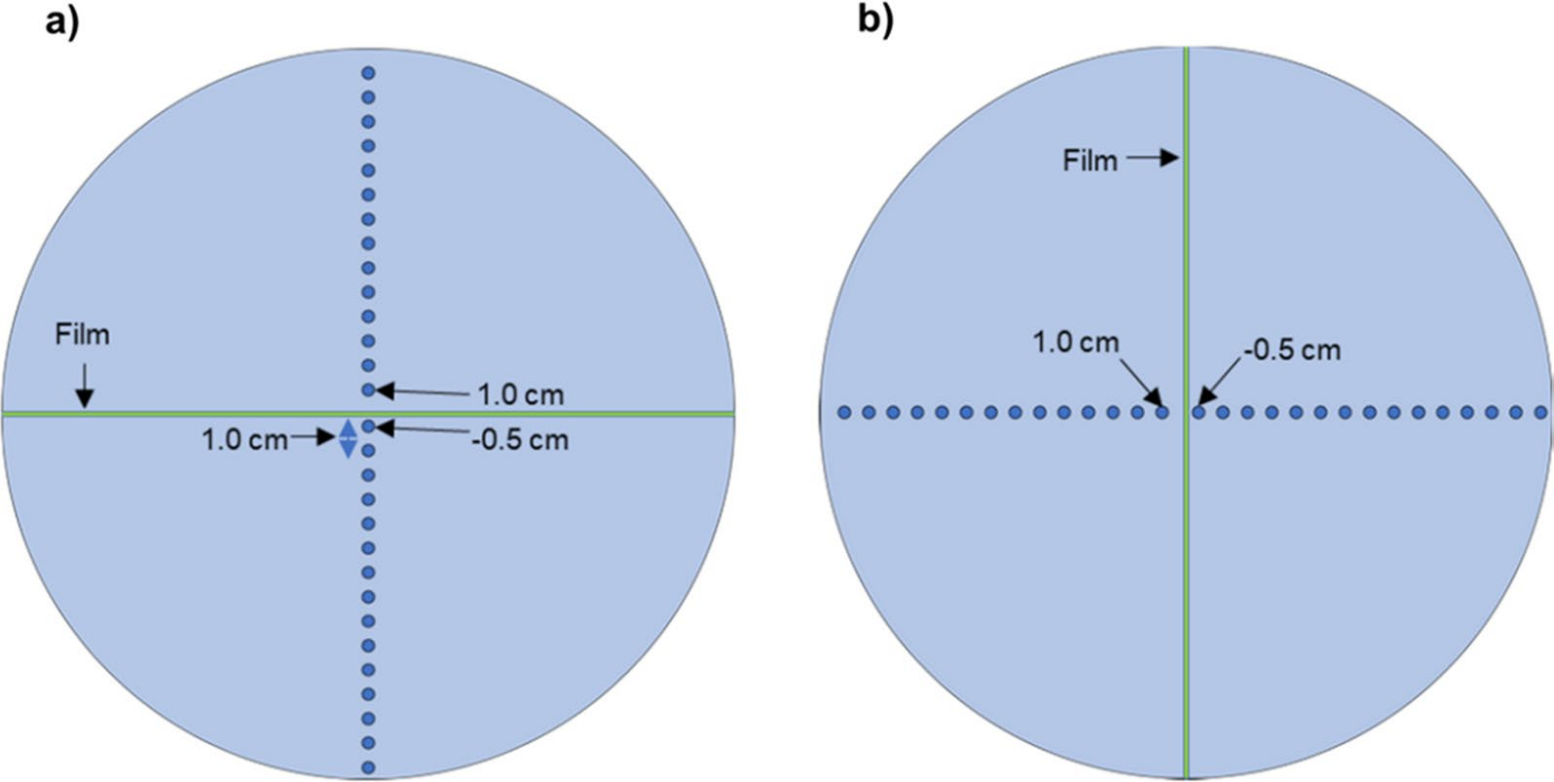
Schematic diagram of the front view of the vendor-supplied Virtual Water^tm^ phantom for dose measurements. Experimental setup with a film inserted in** a** the coronal plane and **b** the sagittal plane

Among the eight cases that satisfied the criteria in the conventional QA method but failed in TomoEQA, only two cases had logical reasons for the failure, namely the point dose difference was observed in the superficial region where the dose calculation of Mobius3D is known to be inaccurate. However, six other cases were classified for further validation of TomoEQA results; therefore, additional measurements were conducted. Considering case#1 as an example, the results of TomoEQA showed under-dosage in the inferior regions at the off-axis, as shown in Figures S-1-1 and S-1-2 (see Additional file 1). In the 3D gamma index map of TomoEQA, cold spots with more than 2% difference in the gamma index values can be observed. A comparison of the dose profiles also showed a similar tendency, and dose differences of approximately 5–7% were observed between the reference and TomoEQA. Similarly, in other cases, over-dosage, under-dosage, or both were observed only in the results of TomoEQA. To measure and validate the areas where hot or cold spots appeared in the results of TomoEQA, we changed the experimental setup of the typical setup for DQA. Depending on the plan, the phantom was placed with a 90° rotation to insert a film on the sagittal plane, the chamber was located far from the isocenter, or both were applied. A schematic diagram of the experimental setup is shown in Fig. 3.

**Fig. 4:**
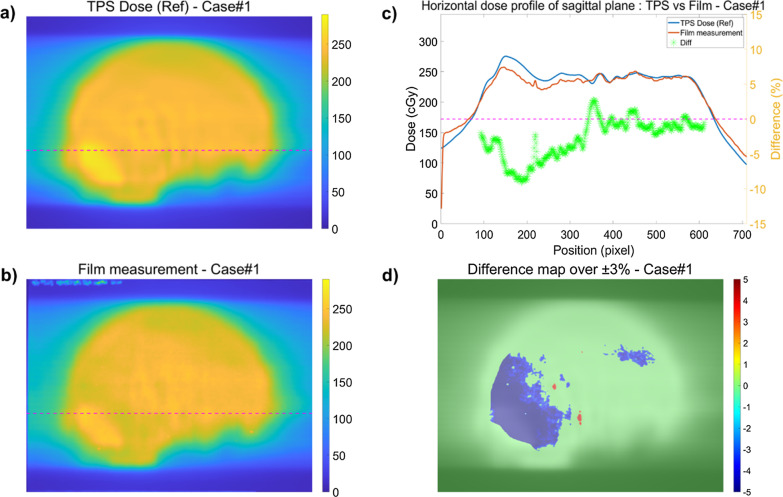
Results of additional measurements with the conventional QA for case#1. 2D dose distribution of **a** reference and **b** film measurement. **c** Comparison of the dose profiles of the reference and film measurement. **d** Dose difference map between the reference and film measurement

The results of additional measurements conducted for the six cases that satisfied the criteria in the conventional QA method but failed in TomoEQA are summarized in Table 5. Figure 4 shows the results of the additional measurements using conventional QA for case#1. For case#1, the phantom was placed with a 90° rotation to measure the dose in the sagittal plane. The 2D dose distributions of the reference and measured films are shown in Fig. 4a and b, respectively, and the comparison of the dose profiles is plotted in Fig. 4c. For simplicity, only the dose difference in the region corresponding to more than 70% of the maximum dose is plotted. Dose differences between the reference and measured films are also plotted in Figure (d); only dose differences of over ± 3% are colored. The result of the re-measurement shows a similar cold spot at the same position as that observed in the results of TomoEQA. In all other cases, the dose differences in TomoEQA were also measured using a similar approach, which revealed the consistency and accuracy of TomoEQA. The results for the other cases from the additional measurement, including the 3D gamma index map of TomoEQA, 2D dose map, line profiles, and difference map, are presented in Additional file 1.

**Table 5:** Summary of the results of re-measurements for six cases requiring further validation

Case	Site	Conventional QA		TomoEQA		Re-measurement			
		Gamma (%)	PD (%)	Gamma (%)	PD (%)	Setup	Chamber position	Gamma (%)	PD (%)
1	Brain	91.48	− 3.56	88.50	− 1.15	(b) Sagittal	− 0.5 cm	81.25	− 1.80
2	Head and Neck	94.74	− 2.19	97.80	− 5.68	(a) Coronal	− 0.5 cm	78.60	− 2.81
3	Brain	93.47	0.30	87.10	0.84	(b) Sagittal	− 4.5 cm	90.08	− 1.85
4	Pelvis	90.64	− 2.50	87.30	− 0.49	(a) Coronal	− 0.5 cm	89.28	− 1.37
5	Pelvis	94.03	− 4.31	78.90	− 6.78	(a) Coronal	− 0.5 cm	92.69	− 3.50
6	Large	96.64	0.42	81.80	1.60	(b) Sagittal	− 0.5 cm	90.45	0.48
7	Brain	97.43	− 1.55	89.40	− 1.97	(b) Sagittal	− 0.5 cm	88.38	− 2.72

*PD* point dose error

### Statistical evaluation of individual plan parameters according to pass/failure of pre-treatment QA

**Fig. 5:**
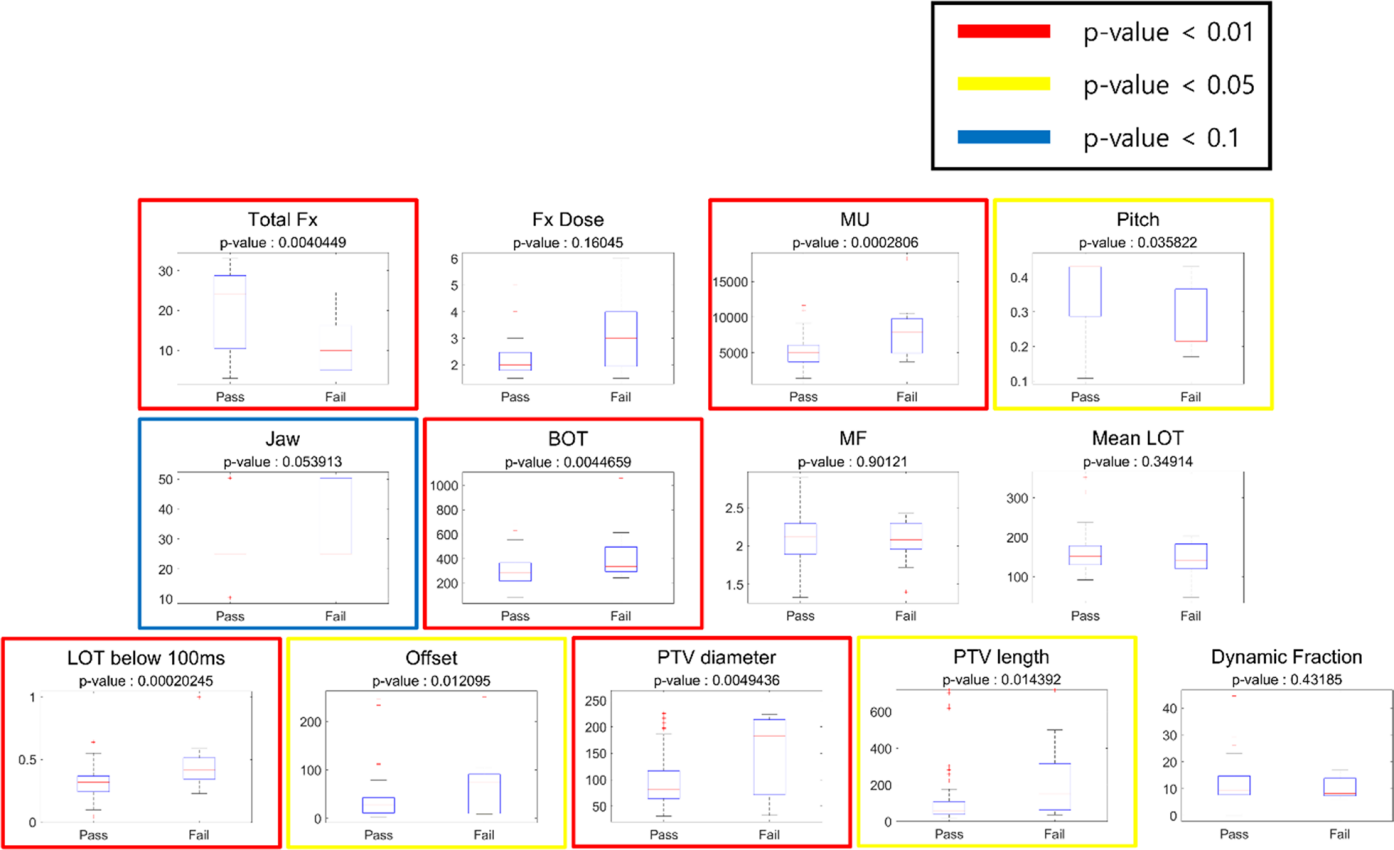
Box plot and *p *values of plan parameters on the pass/failure of pre-treatment QA results using TomoEQA

Statistical analysis was performed for the thirteen plan parameters that are specified in the patient characteristics summarized in Table 2. We investigated whether the pass/failure groups significantly differed based on the individual plan parameters. The box plot diagrams and *p* values of each plan parameter for the pass/failure of the pre-treatment QA results from TomoEQA are shown in Fig. 5. These parameters were classified into three groups according to their significance level expressed in terms of the *p* value, and the corresponding box plot diagrams are highlighted in color. For the parameters “Total Fx,” “MU,” “BOT,” “LOT below 100 ms,” and “PTV diameter,” the *p* value was less than 0.01, and they were classified as “highly significant.” This indicates that there is a significant difference between the pass group and the failure group depending on the value of these parameters. The parameters “Pitch,” “Offset,” and “PTV length,” the *p *value was greater than 0.01 but less than 0.05, and they were classified as “statistically significant.” Finally, the parameter “Jaw” was categorized as “can be significant,” as it had a *p *value greater than 0.05 but less than 0.1, indicating a weaker statistical significance than the other categories. In summary, it was confirmed that nine out of the thirteen plan parameters showed significant differences in the pass/failure of pre-treatment QA results for TomoEQA.

## Discussion

This study performed an in-depth clinical evaluation of TomoEQA, which is an LOT-based patient-specific pre-treatment QA method for HT, by comparing its results with those of the conventional phantom-based method. The results of a comparative study on the data of one hundred patients showed that the QA results of TomoEQA and those of the conventional method are comparable. Although the values were slightly different, TomoEQA satisfied the pass/failure criteria, similar to the conventional method. In addition, some cases that met the criteria in the conventional method but failed in TomoEQA were further investigated by changing the measurement setup, including changing the position of the chamber or the plane on which the film was placed. The results revealed that the results of the additional measurements were consistent with those of TomoEQA.

Cheese phantom-based patient-specific QA methods, measuring point dose and 2D dose distribution by using chamber and film, respectively, were employed as a standard method in HT. There are several issues to be considered when using these strategies in clinical practice. Calibration procedures for the ionization chamber and radiochromic film need to be performed before using them. A careful setup for the measurements is also essential as errors may easily occur depending on the measurement location. In the case of films, sufficient time should be provided for film stabilization to ensure accurate analysis of the measurements; therefore, the results cannot be obtained immediately. Because of the inherent characteristics of the conventional method, a 3D dose distribution cannot be acquired. Therefore, it is difficult to check the dose in the area outside the measurement region, such as a different plane of the off-axis position. In HT, the patient is moved and treated while the beam source rotates, and hence, pitch-dependent ripple-shape dose variations, known as “thread effect” may occur, which adversely affects the dose uniformity on the axial plane [21]. The optimal pitch (expressed as 0.86/n) that can minimize pitch dependence has been empirically proposed in various studies [21–24]. Nevertheless, there may still be a difference in the dose uniformity. Moreover, the aforementioned dose error due to the thread effect is expected when the treatment target is too far from the beam central axis or when the treatment plan includes multiple scattered targets. Therefore, it is critical to validate the dose in the different planes of the off-axis position when performing patient-specific QA for HT.

Because of the difficulties experienced in the existing method while handling this issue, TomoEQA can be a reliable alternative. TomoEQA can provide a 3D dose distribution, and hence can detect dose errors better than the conventional method, especially in the off-axis position. In this study, there were some cases where a dose error not detected by the conventional method was detected only in TomoEQA. Additional measurements determined that an error that could not be detected using the existing method occurred, and it was confirmed that TomoEQA can provide reliable results and be an alternative to the QA method for HT.

One of the limitations of this work is that the results of film dosimetry lacked careful considerations to reduce the effects of various physical factors [25–27]. The positional effect of the scanner was evaluated using a blank film, and it was confirmed that the maximum error was within 0.25%; therefore, a non-uniformity correction for the positional effect of the scanner was not applied. However, a dose-dependent non-uniformity was not evaluated and corrected in this study; therefore, a nonnegligible error may occur in the edge region, especially in large fields. The air gap between the phantom and film is another factor that can affect the results. A phantom with a 90° rotation was employed to examine the dose in the sagittal plane. However, the measurement results may have been adversely affected by the air gap as this is a setup where the air gap can easily occur. Overall, these physical factors may perturb the film dosimetry results. Therefore, to obtain more robust measurement results, we envision that one can conduct additional experiments using array-based devices in the future.

The plan parameters of HT that have a statistically significant influence on the pass/failure of pre-treatment QA were examined in conjunction with TomoEQA. The results are relatively consistent with those of previous research [28–31]. The findings of this study, including dose inaccuracy in the off-axis region, are considered to be a result of the thread effect.

TomoEQA is convenient for clinical application because it does not require phantoms, chambers, or films, which are used in the conventional method. Instead, TomoEQA only requires exit detector data, which can be acquired through plan delivery without a phantom. Similarly, human errors are inevitable in the setup of the conventional method, measurements, and analysis procedures. In addition, there exists inter-variability between operators, which adversely affects patient-specific QA results. However, TomoEQA can provide consistent results without human error and inter-variability between workers because there is no potential for the error to be involved in its workflow.

The accuracy of beam modeling affects the results of TomoEQA and dose calculation algorithm of the TPS utilized, and hence requires detailed understanding and consideration. In Mobius3D, which is employed in the TomoEQA workflow, the user cannot tune the beam data and relevant modeling of the HT system, except the output factor and dosimetric leaf gap correction factor. Therefore, the accuracy of dose calculation in TomoEQA is nearly impossible to improve unless the vendor provides an update in the future. The beam data implemented in the beam model of the TPS and the current beam condition of the system must be thoroughly matched, and beam-related parameters, including energy, output, and symmetry, should be managed. In addition, because the actual LOT is derived from data measured using the exit detector, the conditions of the exit detector, which may affect the QA results, should also be maintained at an optimal level. It is recommended to check for the presence of dead pixels and the signal response for each detector module, which can be monitored through periodic imaging and mechanical QA for the detector. Replacing insufficient detector data that may occur in dynamic jaw delivery with planned data (mentioned in the Method section) is also one of the factors that can affect the QA results. Therefore, it is also necessary to evaluate the degree of similarity between partially replacing the detector data with planned data and the actual true data, which will be investigated in our future study.

Another limitation is that unlike Precision, the primary TPS of the Accuray system and other commercially available TPSs may not reflect the physical properties of the binary MLC of HT, such as MLC latency. In addition, the accuracy of dose calculation may be relatively low as Mobius3D utilizes a simplified version of the dose calculation algorithm (simplified collapsed cone convolution algorithm) compared with that used in the primary TPS. Therefore, these obstacles are difficult to overcome as they require significant improvement in the TPS in TomoEQA. Similarly, the inaccuracy of dose calculation for small fields in secondary TPS, including Mobius3D, is another factor that affects the pre-treatment QA result. These factors were considered in the results of TomoEQA in this study, and it was observed that the dose in some regions tended to be either overestimated or underestimated. Further investigations confirmed that TomoEQA overestimated or underestimated the dose in some hot and cold spots.

Nevertheless, it is expected that significant problems will not occur while using TomoEQA in clinical applications for patient-specific pre-treatment QA in HT systems. Mobius3D, which was used in this study for dose calculation in TomoEQA, was originally a solution for pre-treatment QA in general LINAC systems. In our institute, pre-treatment QA has been performed for patients who were treated using a 6 MV beam with a LINAC system using Mobius3D with a passing rate of approximately 94%. Considering that Mobius3D does not currently support fine-tuning of the HT system, the passing rate of TomoEQA, which corresponds to 91%, is believed to be sufficient compared with those used in other clinical practices.

## Conclusions

In our previous work, TomoEQA, a log-based patient-specific pre-treatment QA method dedicated to HT systems, was used to compensate for the shortcomings of conventional QA methods. In addition to the feasibility study on TomoEQA conducted in our previous study, an in-depth clinical evaluation of TomoEQA was performed in the present study using the data of one hundred patients. A comparative analysis was conducted to evaluate the clinical performance of TomoEQA in comparison with that of the conventional method. Furthermore, additional measurements were conducted under other conditions to validate the off-axis region.

It was confirmed that TomoEQA is a clinically applicable alternative to conventional QA methods. This enables users to compare 3D dose distributions, which provides more detailed information than the conventional QA method. In particular, dose errors in the off-axis plane can be detected.

Our plans for future work include a study with more patient cases to ensure a more robust statistical analysis. This is to ensure that the correlation between the plan parameter and pass/failure of pretreatment QA can be accurately determined. We also aim to generate a decision model that can predict pass/failure using only plan parameter information.

## Supplementary Information

**Additional file 1.** The results of additional measurements using the conventional QA for the cases that satisfied the criteria in the conventional QA method but failed in TomoEQA.

## Data Availability

Data availability is limited due to institutional data protection law and confidentiality of patient data.

## Abbreviations

QA: Quality assurance
HT: Helical tomotherapy
Fx: Fraction
MU: Monitor units
BOT: Beam-on-time
LOT: Leaf-of-time
PTV: Planning target volume
LINAC: Linear accelerator
MLC: Multi-leaf collimator
TPS: Treatment planning system
GPR: Gamma passing rate
AD: Absolute dose error
SD: Standard deviations
PD: Point dose error
