# Supplementary material for: Comprehensive clinical evaluation of TomoEQA for patient-specific pre-treatment quality assurance in helical tomotherapy
Source: Radiat Oncol. 2022 Nov 7;17:177. doi: 10.1186/s13014-022-02151-x (PMC9641889; doi:10.1186/s13014-022-02151-x)
Supplement: Supplementary file 1 — Additional file 1. The results of additional measurements using the conventional QA for the cases that satisfied the criteria in the conventional QA method but failed in TomoEQA. [file 13014_2022_2151_MOESM1_ESM.docx]

**Supplementary data** < The results of additional measurements using the conventional QA for the cases that satisfied the criteria in the conventional QA method but failed in TomoEQA>


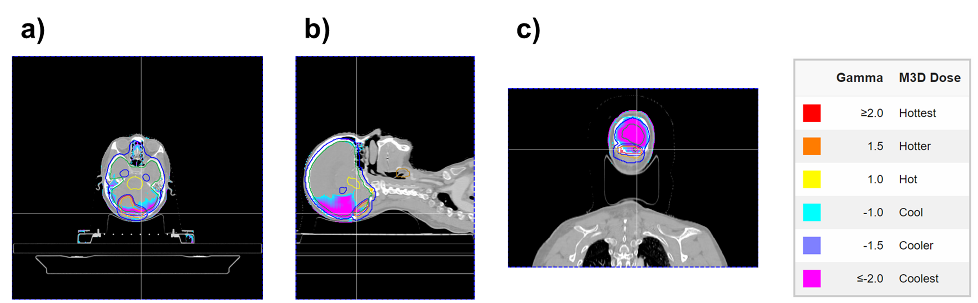


Figure S-1-1. 3D gamma index map of case#1 resulting from TomoEQA in a) transverse, b) sagittal, and c) coronal planes


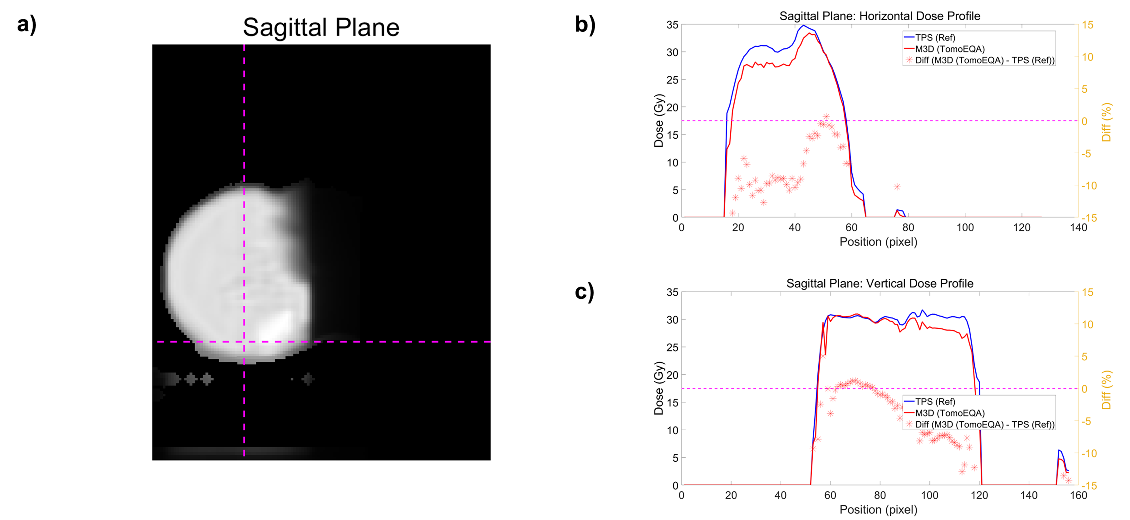


Figure S-1-2. 2D dose distribution of case#1 calculated by TomoEQA in a) sagittal plane and comparisons of dose profiles between TPS and TomoEQA in b) the horizontal and c) the vertical directions.


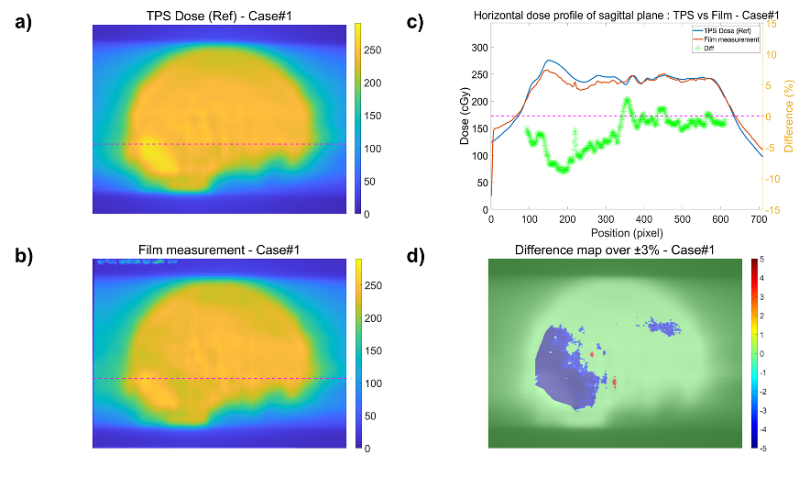


Figure S-1-3. Results of additional measurements with conventional QA for case#1 in the sagittal plane. 2D dose distribution of a) reference and b) film measurements. c) Comparison of dose profiles between reference and film measurements. d) Dose difference map between the reference and film measurements.


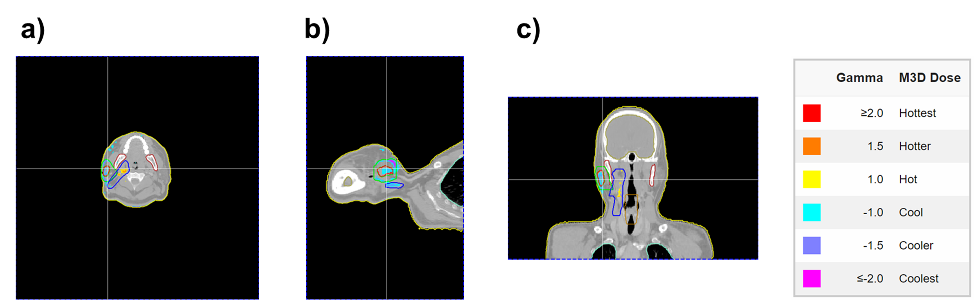


Figure S-2-1. 3D gamma index map of case#2 resulting from TomoEQA in a) transverse, b) sagittal, and c) coronal planes


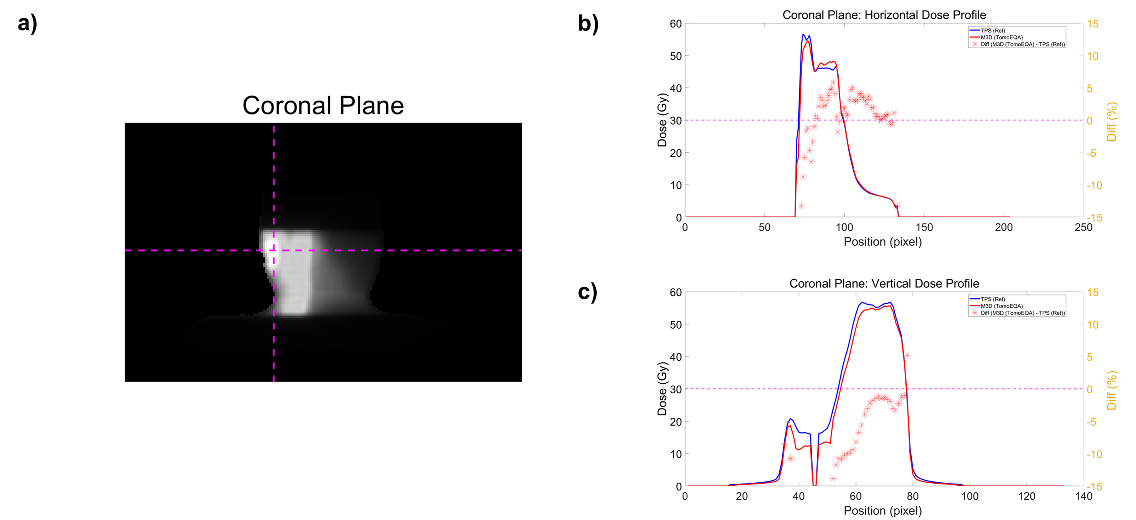


Figure S-2-2. 2D dose distribution of case#2 calculated by TomoEQA in a) coronal plane and comparisons of dose profiles between TPS and TomoEQA in b) the horizontal and c) the vertical directions.


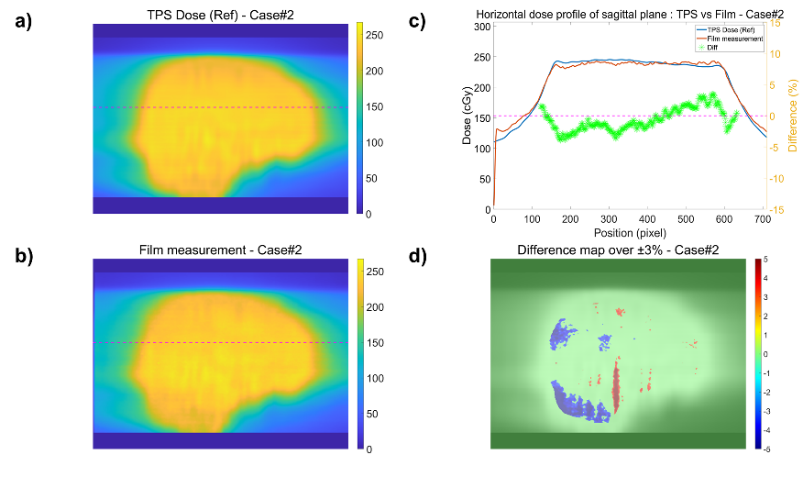


Figure S-2-3. Results of additional measurements with conventional QA for case #2 in the coronal plane. 2D dose distribution of a) reference and b) film measurements. c) Comparison of dose profiles between reference and film measurements. d) Dose difference map between the reference and film measurements.


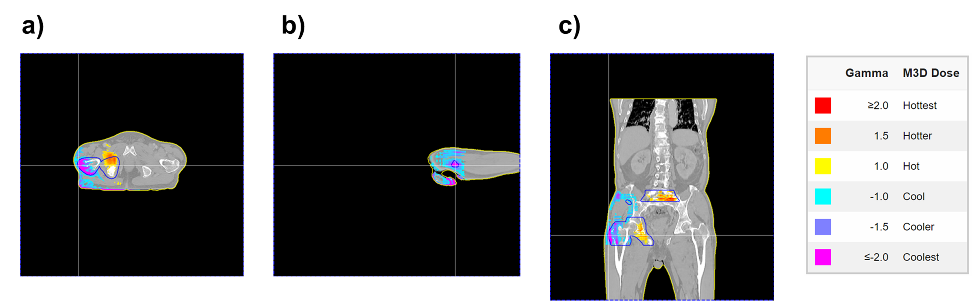


Figure S-3-1. 3D gamma index map of case#3 resulting from TomoEQA in a) transverse, b) sagittal, and c) coronal planes


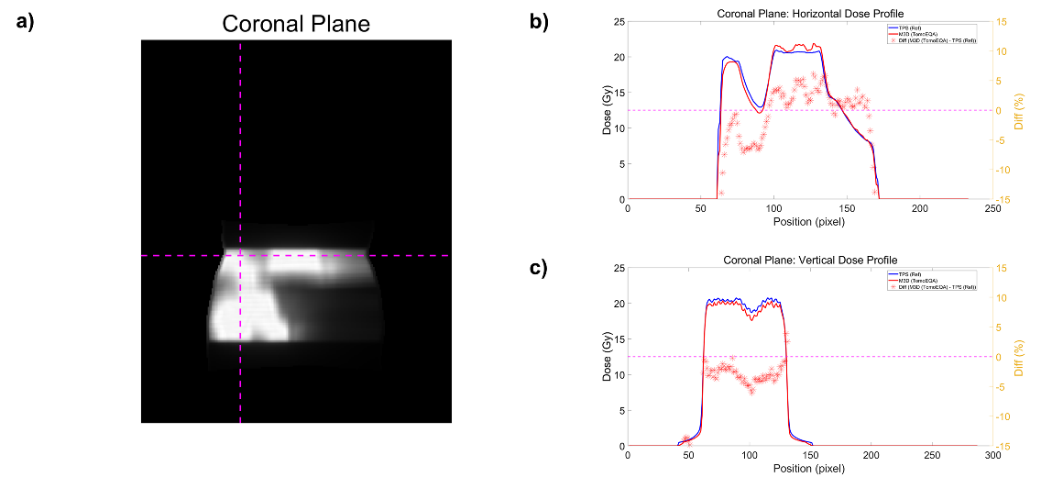


Figure S-3-2. 2D dose distribution of case#3 calculated by TomoEQA in a) coronal plane and comparisons of dose profiles between TPS and TomoEQA in b) the horizontal and c) the vertical directions.


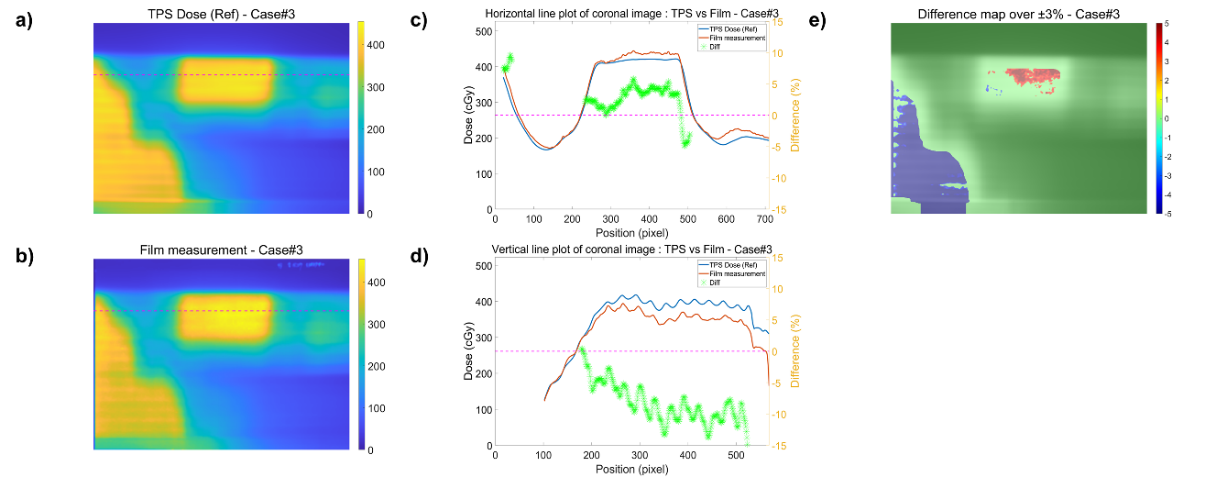


Figure S-3-3. Results of additional measurements with conventional QA for case#3 in the coronal plane. 2D dose distribution of a) reference and b) film measurements. Comparisons of dose profiles between reference and film measurements in the) horizontal and d) vertical directions, respectively. e) Dose difference map between the reference and film measurements.


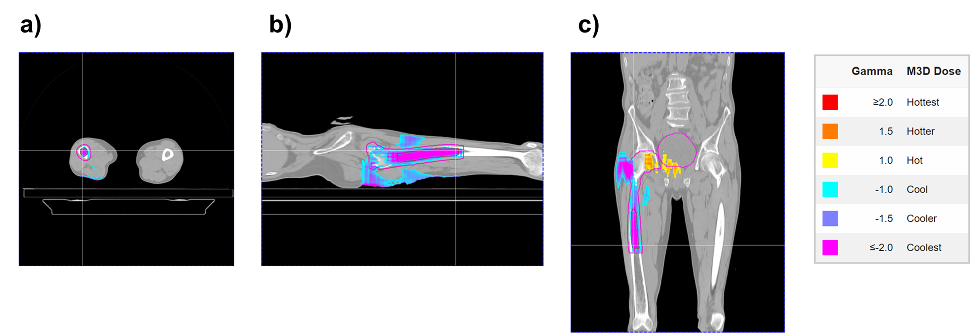


Figure S-4-1. 3D gamma index map of case#4 resulting from TomoEQA in the) transverse, b) sagittal, and c) coronal planes


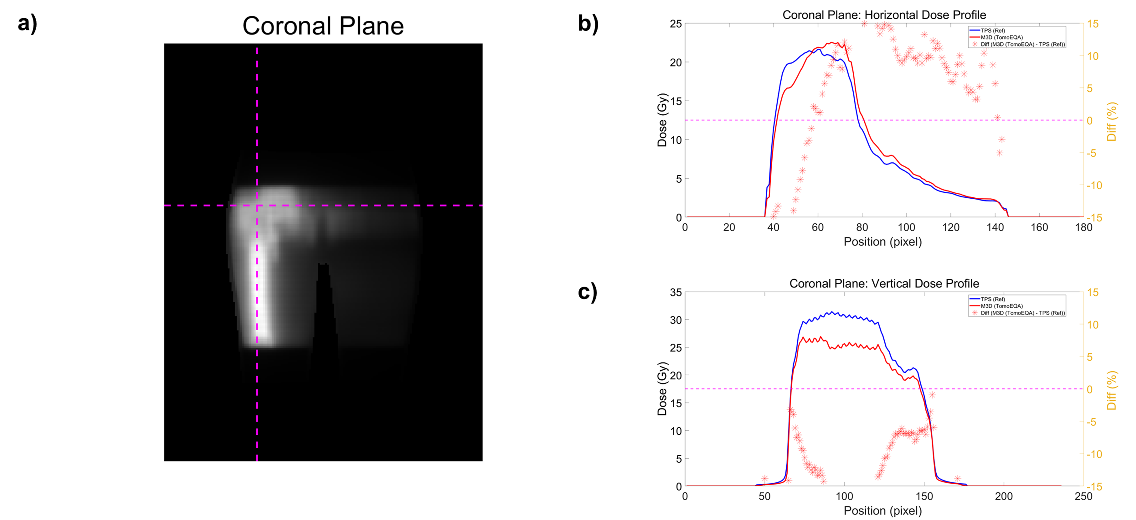


Figure S-4-2. 2D dose distribution of case#4 calculated by TomoEQA in a) coronal plane and comparisons of dose profiles between TPS and TomoEQA in b) the horizontal and c) the vertical directions.


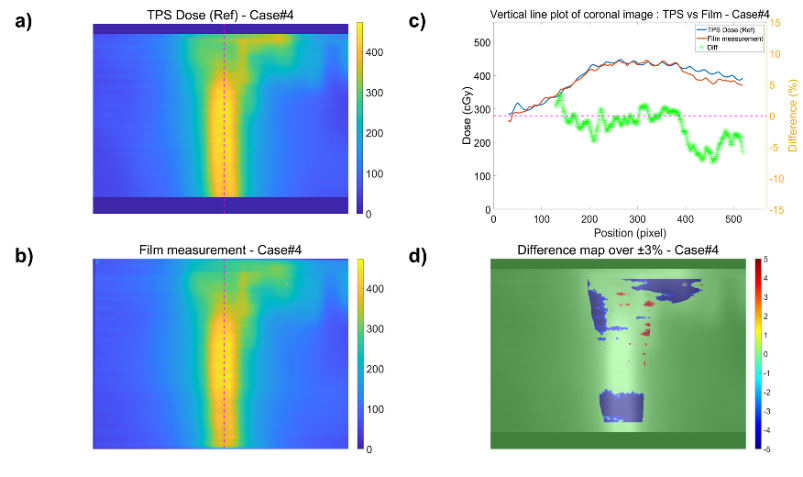


Figure S-4-3. Results of additional measurements with conventional QA for case #4 in the coronal plane. 2D dose distribution of a) reference and b) film measurements. c) Comparison of dose profiles between reference and film measurements. d) Dose difference map between the reference and film measurements.


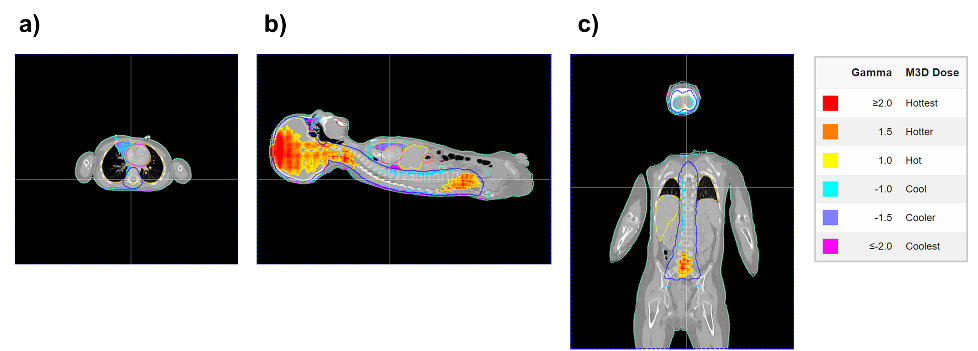


Figure S-5-1. 3D gamma index map of case#5 resulting from TomoEQA in a) transverse, b) sagittal, and c) coronal planes


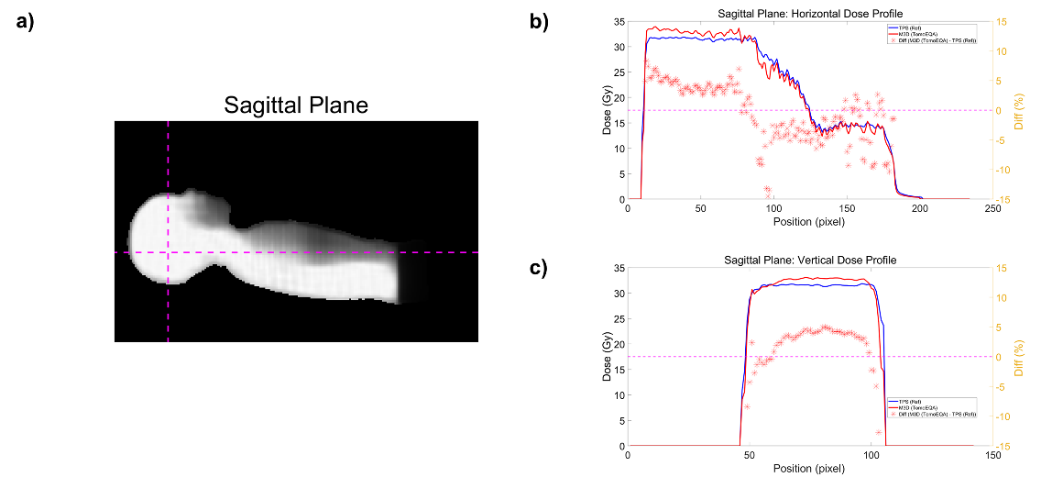


Figure S-5-2. 2D dose distribution of case#5 calculated by TomoEQA in a) sagittal plane and comparisons of dose profiles between TPS and TomoEQA in b) the horizontal and c) the vertical directions.


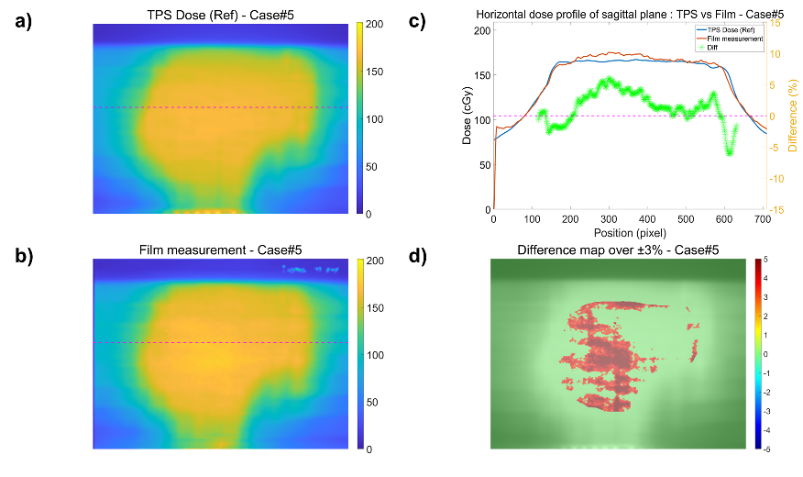


Figure S-5-3. Results of additional measurements with conventional QA for case#5 in the sagittal plane. 2D dose distribution of a) reference and b) film measurements. c) Comparison of dose profiles between reference and film measurements. d) Dose difference map between the reference and film measurements.


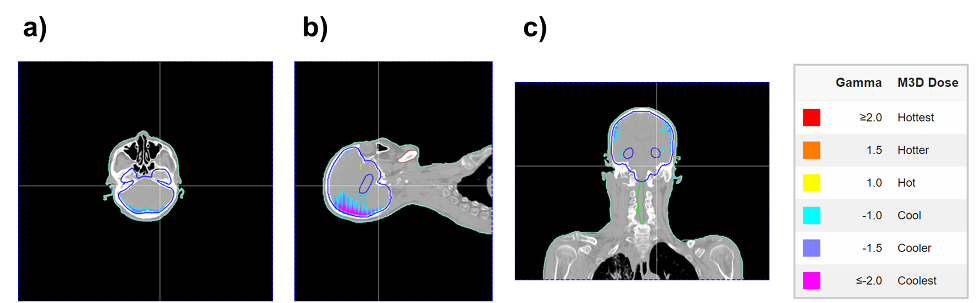


Figure S-6-1. 3D gamma index map of case#6 resulting from TomoEQA in a) transverse, b) sagittal, and c) coronal planes


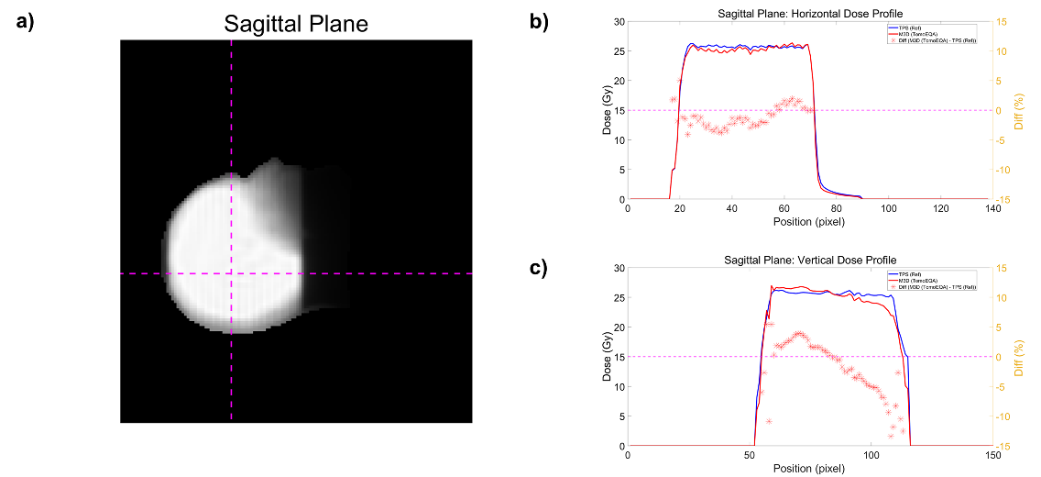


Figure S-6-2. 2D dose distribution of case#6 calculated by TomoEQA in a) sagittal plane and comparisons of dose profiles between TPS and TomoEQA in b) the horizontal and c) the vertical directions.


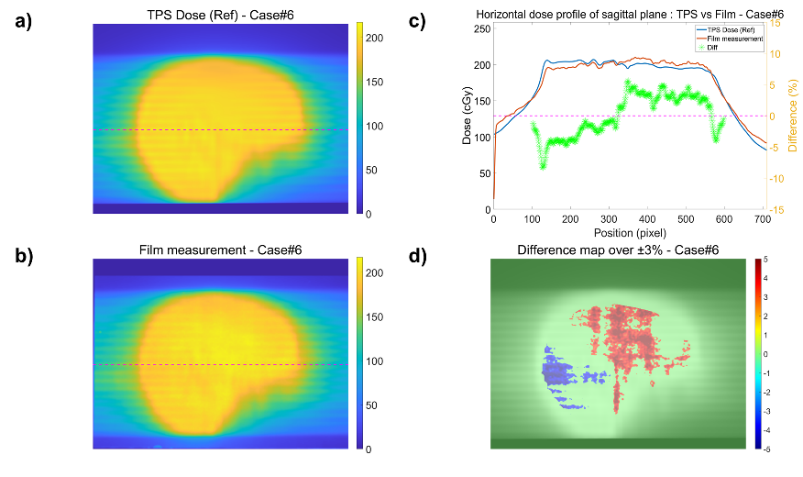


Figure S-6-3. Results of additional measurements with conventional QA for case#6 in the sagittal plane. 2D dose distribution of a) reference and b) film measurements. c) Comparison of dose profiles between reference and film measurements. d) Dose difference map between the reference and film measurements.
